# Uncertainty in techno-economic estimates of cellulosic ethanol production due to experimental measurement uncertainty

**DOI:** 10.1186/1754-6834-5-23

**Published:** 2012-04-17

**Authors:** Kristin J Vicari, Sai Sandeep Tallam, Tatyana Shatova, Koh Kang Joo, Christopher J Scarlata, David Humbird, Edward J Wolfrum, Gregg T Beckham

**Affiliations:** 1 David H. Koch School of Chemical Engineering Practice, Massachusetts Institute of Technology, Cambridge, MA, USA; 2 Department of Chemical Engineering, Massachusetts Institute of Technology, Cambridge, MA, USA; 3 National Bioenergy Center, National Renewable Energy Laboratory, Golden, CO, USA; 4 Department of Chemical Engineering, Colorado School of Mines, Golden, CO, USA

**Keywords:** Pretreatment, Enzymatic hydrolysis, Fermentation, Process modeling, Biochemical conversion, Techno-economic modeling

## Abstract

**Background:**

Cost-effective production of lignocellulosic biofuels remains a major financial and technical challenge at the industrial scale. A critical tool in biofuels process development is the techno-economic (TE) model, which calculates biofuel production costs using a process model and an economic model. The process model solves mass and energy balances for each unit, and the economic model estimates capital and operating costs from the process model based on economic assumptions. The process model inputs include experimental data on the feedstock composition and intermediate product yields for each unit. These experimental yield data are calculated from primary measurements. Uncertainty in these primary measurements is propagated to the calculated yields, to the process model, and ultimately to the economic model. Thus, outputs of the TE model have a minimum uncertainty associated with the uncertainty in the primary measurements.

**Results:**

We calculate the uncertainty in the Minimum Ethanol Selling Price (MESP) estimate for lignocellulosic ethanol production via a biochemical conversion process: dilute sulfuric acid pretreatment of corn stover followed by enzymatic hydrolysis and co-fermentation of the resulting sugars to ethanol. We perform a sensitivity analysis on the TE model and identify the feedstock composition and conversion yields from three unit operations (xylose from pretreatment, glucose from enzymatic hydrolysis, and ethanol from fermentation) as the most important variables. The uncertainty in the pretreatment xylose yield arises from multiple measurements, whereas the glucose and ethanol yields from enzymatic hydrolysis and fermentation, respectively, are dominated by a single measurement: the fraction of insoluble solids (f_IS_) in the biomass slurries.

**Conclusions:**

We calculate a $0.15/gal uncertainty in MESP from the TE model due to uncertainties in primary measurements. This result sets a lower bound on the error bars of the TE model predictions. This analysis highlights the primary measurements that merit further development to reduce the uncertainty associated with their use in TE models. While we develop and apply this mathematical framework to a specific biorefinery scenario here, this analysis can be readily adapted to other types of biorefining processes and provides a general framework for propagating uncertainty due to analytical measurements through a TE model.

## Introduction

Economically-viable production of renewable transportation fuels from lignocellulosic biomass remains a significant technical challenge at the industrial scale. The near-term routes for renewable fuel production include biochemical conversion to ethanol [1-9], pyrolysis [10-12], and gasification [8,13,14], along with newer options that reduce sugars to higher alcohols and hydrocarbon fuels [15-19] or use novel solvents to fractionate the plant cell wall constituents [20-23]. A near-term route to achieve commercially-viable lignocellulosic biomass conversion to ethanol is mild chemical pretreatment followed by enzymatic hydrolysis and fermentation. This route is generally classified as biochemical conversion [2,8]. Within biochemical conversion, there exist multiple options for each of the three primary steps. For example, there are multiple reaction media for the chemical pretreatment step [24-26], including hot water or steam explosion [25,27], dilute sulfuric acid [2], ammonia-fiber explosion [28,29], and lime treatment [30]. In addition, there are several possible process configurations for conducting enzymatic hydrolysis and fermentation simultaneously or separately and with different fermentation organisms [3,7]. These process options are currently under intense development with several plants operating worldwide, and it is not yet known which combinations will be the most economically-viable at scale.

While there is substantial motivation to shift worldwide fuel supplies toward renewable resources, the transition will require enormous investment and development over several decades. To minimize the risk associated with industrial commercialization and deployment of lignocellulosic ethanol and other advanced biofuels, the US Department of Energy has committed significant resources to demonstrate ethanol production via biochemical and thermochemical conversion routes at a price competitive with gasoline at the pilot scale by 2012. A key component of this cost demonstration target is the use of a techno-economic (TE) model of lignocellulosic ethanol production [1]. These types of models typically consist of two complementary parts: a process model and an economic model. TE models are crucial to compare biofuel production options, and have been employed in several comparative analyses conducted to date [5,8,9,11].

In typical biochemical conversion processes for lignocellulosic ethanol [1], the process model consists of three main unit operations: pretreatment, enzymatic hydrolysis, and fermentation. The inputs to the process model are the composition of the feedstock and reaction yields for all reactions in each unit operation. For example, the yield of glucose from cellulose in enzymatic hydrolysis and the yield of ethanol from glucose during fermentation are both inputs in the TE model. These reaction yields are based on the results of laboratory- and pilot-scale experiments and are calculated from measurements of the chemical composition of reactant, intermediate, and product streams. For example, the yield of xylose from xylan during a pretreatment experiment is calculated by measuring the composition of the input biomass feedstock and the biomass slurry after pretreatment. A representative yield expression is illustrative. Equation 1 shows the yield of xylose from a batch pretreatment experiment.

(1) $$Y_{X}=\frac{m_{f}(1-f_{\mathrm{IS}})/\rho_{L}C_{X}}{m_{i}x_{X}}\frac{MW_{\mathrm{xylan}}}{MW_{\mathrm{xylose}}}$$

The numerator of the first term is the mass of soluble xylose in the system, the product of the final slurry mass (biomass plus water plus acid), the mass fraction of soluble solids in the final slurry (converted to volume with the density) and the concentration of xylose in the soluble fraction. The denominator in this equation is the product of the initial mass of biomass and the mass fraction of xylan in the sample; the initial amount of xylan in the system. The second term is a conversion factor for xylose (MW = 150) and xylan (MW = 132). Since each of the measured variables (*m*_*f*_*, m*_*i*_*, x*_*X*_*, fis, r*_*l*_*, C*_*X*_) have some uncertainty, they all contribute to the uncertainty in the yield expression. Overall, the outputs of the process model are mass and energy balances across each unit operation and across the entire biomass-to-ethanol process. The economic model estimates capital and operating costs given the mass and energy balances from the process model and a number of assumptions regarding capital and operating costs. The main output of the TE model is the minimum ethanol selling price (MESP) [1,2], which is defined as the price of ethanol at the plant gate where ethanol revenue balances its production cost.

The uncertainty in MESP for a given TE model is due to uncertainties in both the process model and the economic model. For example, uncertainty in the feedstock composition will propagate to the uncertainty in the mass and energy balances in the process model, which will in turn propagate to the uncertainty in the economic model. Conversely, uncertainty in the capital costs of the process equipment will directly yield uncertainty in the total plant capital cost in the economic model. Because there are few lignocellulosic ethanol production facilities in the world, estimating the uncertainty in capital and operating expenses is likely to be unreliable, making it difficult to quantify the impact on MESP uncertainty. However, there has been significant work to date regarding the other two contributors to MESP uncertainty: feedstock composition [31-34] and process measurements from pilot-scale operation at "National Renewable Energy Laboratory (NREL)" [35-38]. *Here we quantify the lower bound on the MESP uncertainty for a biochemical conversion process by propagating the effects of uncertainty from feedstock composition and primary measurements to the uncertainty in the economic model. We focus only on the process model portion of the TE model, leaving uncertainty in the economic assumptions for later work.*

The manuscript is organized as follows: first, we describe the process model examined here, which is taken from the NREL 2011 Biochemical Conversion Design Report [39]. We discuss the origin of the experimental data used in this work. We define absolute and relative uncertainties. We then convert the yield calculations for pretreatment, enzymatic hydrolysis, and fermentation to analytical relationships from which the uncertainty expressions are developed. Using these relationships, we describe the calculations for yield uncertainties for the important reactions in each unit operation and present the fractional contribution of each input uncertainty to the yield uncertainty. We then describe the Monte Carlo simulations conducted to quantify the lower bound on precision in MESP propagated from the yield uncertainties from the process model. Finally, we discuss the utility of this approach and its generality for estimating cost uncertainties in TE models for lignocellulosic biomass conversion to fuels.

### Process model

The process model used for this study is from the 2011 Design Report for biochemical conversion of lignocellulosic biomass to ethanol [39]. We examine the feedstock composition and three unit operations: a mild chemical pretreatment with dilute sulfuric acid, enzymatic hydrolysis, and co-fermentation of pentose and hexose sugars to ethanol.

### Feedstock composition

The feedstock composition includes structural carbohydrates (glucan, xylan, arabinan, mannan, galactan), lignin, soluble carbohydrates, ash, protein, and extractable materials not otherwise specified. The structural and soluble carbohydrates are used to produce ethanol. The lignin in the feedstock is burned to provide heat and power. The protein, ash, and unspecified extractable material are included to complete the mass balance but are not used to produce ethanol or energy.

### Pretreatment

The pretreatment unit converts hemicellulose into soluble monomers and oligomers via dilute sulfuric acid hydrolysis. The products are primarily monomeric and oligomeric xylose, arabinose, galactose, and a small amount of glucose. The idealized reaction scheme in the pretreatment unit consists of 21 reactions [39]. Only 5 of these reactions involve xylan, three of which produce xylose; the other two produce undesirable byproducts. The remaining 16 reactions involve the conversion of the other structural carbohydrates to oligomeric and monomeric sugars and unwanted byproducts. The required inputs to this unit operation are the composition of the biomass feedstock, the flowrates of feedstock and dilute acid, and the yields of all 21 reactions.

### Enzymatic hydrolysis

In the process model, the enzymatic hydrolysis unit follows the pretreatment unit. The primary reactions in the enzymatic hydrolysis unit are the conversion of cellulose to cellobiose (a glucose dimer) and cellobiose to glucose. The idealized reaction scheme in the enzymatic hydrolysis unit consists of 4 reactions occurring in each of two reactor vessels in series. Thus, the model includes a total of 8 reactions, all of which involve cellulose or cellobiose [39]. The required inputs to this unit operation are the yields of the 8 reactions. The composition of the pretreated slurry entering the reactor is calculated in the previous unit operation.

### Fermentation

The fermentation unit follows the enzymatic hydrolysis unit and converts the sugar monomers to ethanol. The idealized reaction scheme in the process model for the fermentation unit consists of 31 different reactions involving five different sugars: glucose, xylose, arabinose, mannose, and galactose. Five of these reactions produce ethanol, while the other 26 reactions produce either undesirable side products (lactic acid, succinic acid) or are used by the fermentative organism to produce additional cell mass. The required inputs to this unit operation are the yields of all 31 reactions [39].

### Experimental data

The compositional analysis data are from recent studies discussing the history and typical uncertainties of standard biomass compositional analysis methods [33,34]. Experimental data for pretreatment are from a 1-ton-per-day continuous-flow pretreatment reactor (unpublished results). Experimental data for enzymatic hydrolysis and fermentation are from batch laboratory-scale experiments (unpublished results). The experimental data used in this analysis are provided in full in the Supplementary Material (Additional File 1).

## Results

**Figure 1:**
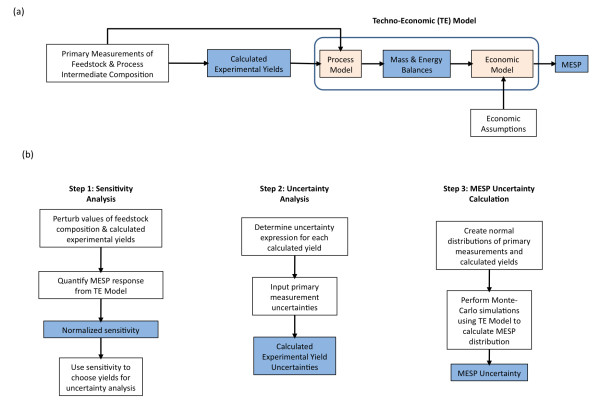
**(a) Summary of the techno-economic (TE) model used in this study.** Primary measurements of feedstock and process intermediate composition are used to calculate experimental yields for each unit operation. These calculated yields as well as the feedstock composition are used in the process model to determine mass and energy balances which are in turn used by an economic model (along with economic assumptions) to calculate the minimum ethanol selling price (MESP). **(b)** Detailed methodology to calculate MESP uncertainty. In Step 1, we determine the normalized sensitivity by quantifying the normalized response in MESP to changes in each variable. These sensitivity values are used as inputs to the uncertainty analysis in Step 2 to determine the uncertainties in calculated yield values based on pilot- and laboratory-scale experimental data. In Step 3 we perform Monte Carlo simulations of the TE model using distributions of the key variables based on the uncertainties calculated in Step 2. Calculated values are shown in blue.

The overall methodology in this study is shown in Figure 1. A process model, developed in Aspen Plus (Burlington MA), is used to integrate simulations of the three main unit operations. Within each unit, the model calculates mass and energy balances based on a slate of possible reactions and yield values, which are based on experimentally-determined yields. To simplify and focus the uncertainty analysis, we have performed a preliminary sensitivity analysis to determine how the MESP value changes in response to changes in each conversion. This analysis highlights those conversions whose uncertainty is most important to the overall model. The results of the sensitivity analysis motivate the derivation of analytical expressions to calculate the uncertainty of only those yields that have proven (in terms of sensitivity) to be important for precise calculation of MESP. Finally, the set of yield uncertainties is used in a Monte Carlo analysis of the MESP to provide an estimate of the uncertainty in calculated MESP values due to uncertainty in feedstock composition and primary measurements.

### Selection of reactions and yields from sensitivity analysis

**Figure 2:**
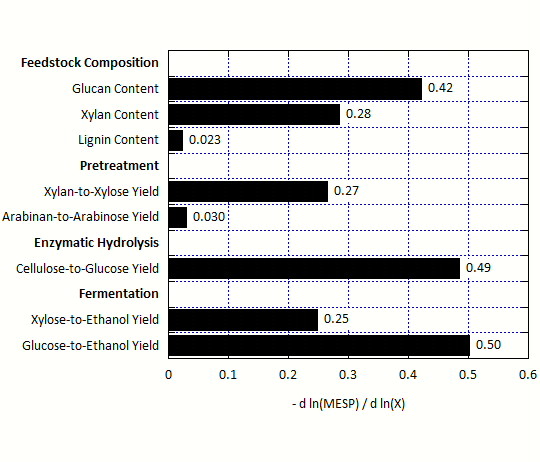
**Normalized sensitivity of minimum ethanol selling price (MESP) to key variables in the process model: biomass composition and reaction yields in pretreatment, enzymatic hydrolysis, and fermentation.** All values are negative, indicating that the MESP decreases when feedstock composition and reaction yields increase.

The process model has a total of 64 variables associated with feedstock composition and reaction yields in the three unit operations: 14 for composition, 21 for pretreatment, 8 for enzymatic hydrolysis, and 31 for fermentation. Preliminary sensitivity analyses were performed for all of these variables using an older version of the process model [1]. The results identify a small set of composition and yield variables with the greatest effect on the MESP value. We performed sensitivity analyses for just these variables using the current process model [39]. Because the primary differences in the two process models are centered on updates to reactor design choices, pretreatment neutralization strategy, and wastewater treatment design, we anticipate that there will be little to no differences in the primary variables that affect MESP uncertainty. Figure 2 shows the results of the second set of calculations, delineated by unit operation. All values of normalized sensitivity for these variables are negative, meaning that an increase in the variable results in a decrease in MESP.

The glucan and xylan content of the feedstock are the most important composition variables, with lignin being much less important. In the pretreatment step, the yield of xylose from xylan is most important, with the yield of arabinose from arabinan a distant second. In the enzymatic hydrolysis step, the yield of glucose from cellulose is most critical, and the yields of ethanol from the major sugars glucose and xylose are the most important in the fermentation step.

### Results of uncertainty analysis

We performed an uncertainty analysis for the five yield expressions identified from the sensitivity analysis and presented in Figure 2: xylose from xylan and arabinose from arabinan in pretreatment, glucose from cellulose in enzymatic hydrolysis, and ethanol from glucose and xylose in fermentation.

**Figure 3:**
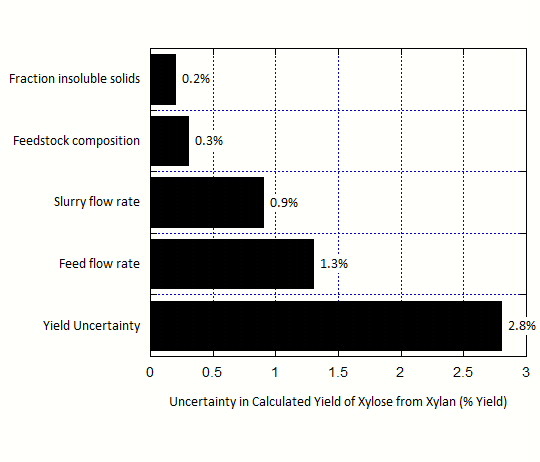
**Contributions to the uncertainty in the calculated yield of xylose from xylan based on pilot-scale pretreatment data.** The uncertainty in the measured feed flow contributes approximately half of the total uncertainty, with measurements of the slurry flow, feedstock composition, and fraction insoluble solids (f_IS_) contributing the other half.

For the pilot-scale experimental pretreatment data, the calculated yield of xylose from xylan is 56.4 %, and the uncertainty is calculated to be 2.8 %. Thus the yield can be reported as 56.4 ± 2.8 %. Figure 3 shows the contribution of the key primary measurements to the total uncertainty. The main contributors to uncertainty are fraction insoluble solids (f_IS_) of the pretreated slurry, the feedstock flow rate, the pretreated slurry flow rate, and the xylan and glucan content in the feedstock. The uncertainty in these four measurements drives over 90 % of the overall uncertainty. Of these four process variables, f_IS_, the feed flow rate, and the feedstock composition are directly measured, while the slurry flow rate is derived from other measurements. Further details can be found in the Supplementary Material (Additional File 2).

**Figure 4:**
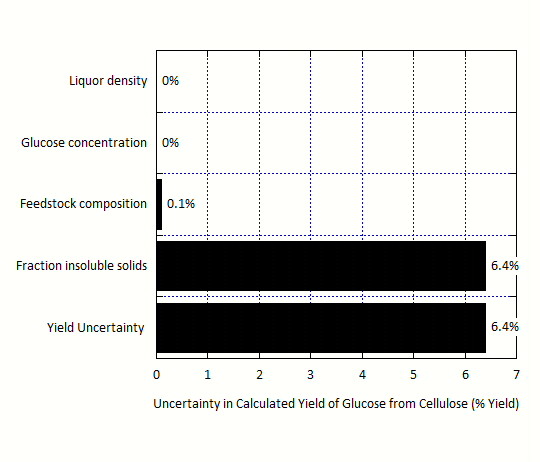
**Contributions to the uncertainty in the calculated yield of glucose from cellulose based on laboratory-scale enzymatic hydrolysis data.** The uncertainty in the calculated glucose yield is almost completely driven by the uncertainty in the fraction insoluble solids (f_IS_) measurement.

For the laboratory-scale enzymatic hydrolysis data, the calculated yield of glucose from cellulose is 77.8 % with an uncertainty of 6.4 %, such that the conversion can be reported as 77.8 ± 6.4 %. Figure 4 shows that this uncertainty is overwhelmingly driven by the uncertainty in the f_IS_ measurement. The uncertainties in the feedstock composition, glucose concentration in the liquor, and liquor density are much less important. Further details can be found in the Supplementary Material (Additional File 3).

**Figure 5:**
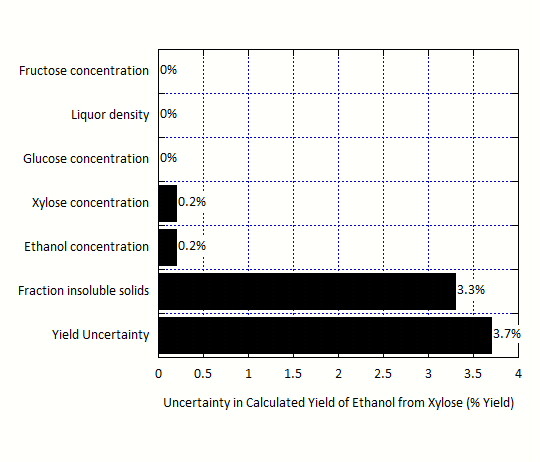
**Contributions to uncertainty in the calculated yield of ethanol from xylose based on laboratory-scale fermentation data.** The uncertainty in ethanol yield is almost completely driven by the uncertainty in the fraction insoluble solids (f_IS_) measurement.

For the laboratory-scale fermentation data, the calculated yields of ethanol from the major sugars glucose and xylose as well as the minor sugar fructose are not independent. Since sugars can be converted to ethanol, undesirable byproducts, or cell mass, it is impossible to determine specific ethanol yields for each sugar. The values for yield of ethanol from glucose and fructose are assumed to be 95 %, and the ethanol yield from xylose is calculated based on this assumed value and knowledge of the beginning and ending concentrations of ethanol and the sugars. Furthermore, the current model does not account for the yields of ethanol from galactose or arabinose. Therefore, the uncertainty analysis has been performed only for the xylose-to-ethanol reaction, with the understanding that the resultant uncertainty also applies to the yield for the glucose-to-ethanol reaction. The calculated yield of xylose to ethanol based on the laboratory data is 86.2 % with an uncertainty of 3.7 %, and thus can be reported as 86.2 ± 3.7 %. The decomposition of this uncertainty into its components reveals that the f_IS_ measurement is the main driver of this uncertainty, as shown in Figure 5. The uncertainty in the concentrations of glucose, xylose, fructose, and ethanol are much less important. Further details can be found in the Supplementary Material (Additional File 4).

### MESP uncertainty – Monte Carlo simulation results

**Figure 6:**
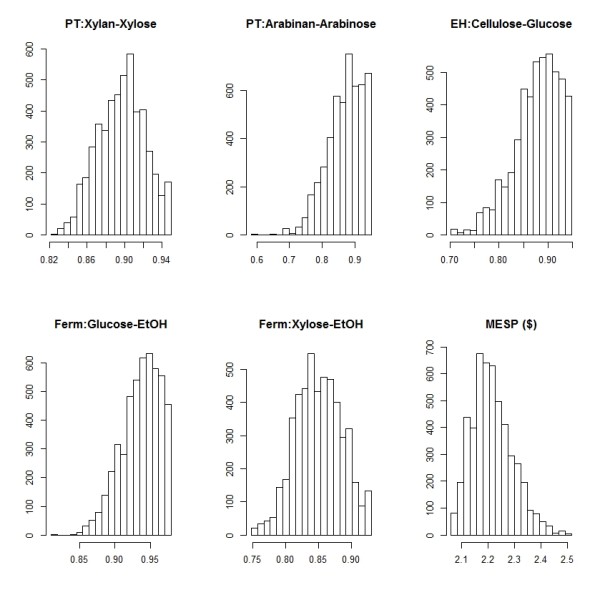
**Distribution of input yield variables and calculated minimum ethanol selling price (MESP) values from the Monte Carlo analysis of the techno-economic (TE) model.** A total of 5000 runs were performed. The mode of the distribution is $2.17/gal and the 95 % confidence interval is $2.09-2.39/gal. Thus, the uncertainty in the MESP calculation caused by the uncertainty in primary measurement variables is half this range, or $0.15/gal.

**Figure 7:**
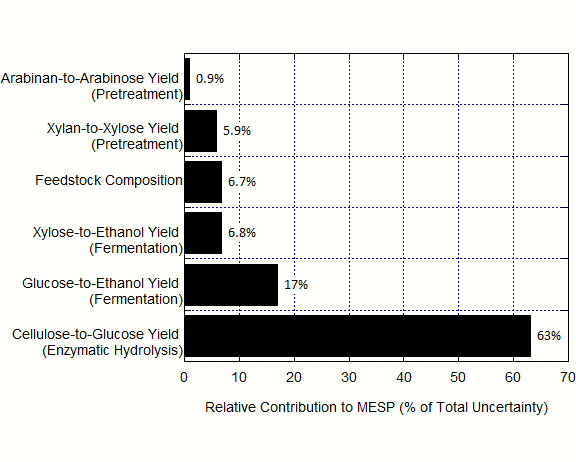
**Contributions to uncertainty in the estimate of minimum ethanol selling price (MESP).** Uncertainty in fermentation yields contributes approximately 30 % to the total uncertainty; uncertainty in enzymatic hydrolysis contributes approximately 60 % to the total uncertainty; and uncertainty in pretreatment and feedstock composition contribute approximately 10 %.

The Monte Carlo results summarize a total of 5,000 Aspen Plus simulation runs that sample parameters from normal distributions of the yields examined in the uncertainty analysis. The baseline values are from the NREL 2011 Biochemical Conversion Design Report [39]. The standard deviations are set equal to the uncertainties calculated in this work. The distributions of the input variables and the calculated MESP values are shown in Figure 6. Some of the input yield distributions are distinctly asymmetric, since the maximum values of these yields were fixed. The resulting MESP distribution is more symmetric, but still positively skewed. The distribution has a mode of $2.17/gal and a mean value of $2.21/gal. The mode value is in excellent agreement with the 2011 Design Report value of $2.15/gal. We calculated a probability interval (p = 0.95) of the resulting MESP distribution to be $2.09-$2.39/gal. Using principal component analysis, we apportioned this uncertainty among the three unit operations as follows: 30 % apportioned to fermentation, 60 % to enzymatic hydrolysis, and 5 % each to pretreatment and feedstock composition (Figure 7).

## Discussion

Our results highlight the central concept of this type of analysis: uncertainties in input parameters to models can have widely varying impacts on the uncertainties in the model outputs. The results of each combined sensitivity and uncertainty analysis can guide the future development of individual unit operation models and the overall process model. From this study, we can highlight the measurements most crucial to the development of a precise estimate of product conversions and, to a certain extent, of plant cost.

### Sensitivity analysis

The results of the sensitivity analysis summarized in Figure 2 are as expected. The key feedstock composition variables are the major structural carbohydrates glucan and xylan, which are the major plant cell wall components converted to ethanol. Increasing the amount of either of these carbohydrates causes a decrease in MESP. MESP is particularly sensitive to these components because they both directly impact the amount of ethanol produced from the feedstock. The lignin content is much less critical; the process model uses the lignin to produce steam and electricity, but these co-products are much less valuable than ethanol. The most important calculated yield variables are xylose from pretreatment, glucose from enzymatic hydrolysis, and ethanol from glucose and xylose in fermentation.

While both glucan and xylan are converted to ethanol, the MESP is approximately 1.5 times more sensitive to glucan than it is to xylan. The difference in sensitivity is likely caused by three key factors. First, the ethanol content of glucose is two, meaning that one mole of glucose produces two moles of ethanol. The ethanol content of xylose is only 1.67. Second, the pretreatment conversions of glucan to glucose and xylan to xylose are different. Third, the fermentation conversions of the monomers are not the same. As a result of these factors, a 1 % change in the glucan content changes the ethanol production by a larger magnitude than a 1 % change in the xylan content.

### Uncertainty analysis

The results for the pretreatment unit show no extreme values in terms of relative uncertainties and there is no single measurement that overwhelms the uncertainties of the calculated yield values. For the enzymatic hydrolysis and fermentation units, however, the uncertainty in the f_IS_ measurement dominates the uncertainty in calculated yield values. This measurement is defined as the mass fraction of the slurry that is solid; it differs from the “total solids” measurement in that it excludes all components soluble in the water phase; for biomass slurries, the soluble “solids” are principally dissolved carbohydrates (e.g., glucose, xylose, and minor sugars). For dilute acid pretreated biomass slurries, typical values of f_IS_ are 15-30 %. The f_IS_ value of biomass slurries historically has been measured in multiple ways, and a recent publication estimated the uncertainty in this measurement to be approximately 3.0 % by comparing the results from two different methods [40]. This suggested magnitude of the f_IS_ uncertainty, however, is inappropriately large. Subsequent work by our group has shown that the uncertainty in the f_IS_ measurement is no more than 1.0 %. This revised estimate includes contributions from both sample collection and sample analysis (data not shown). This work uses a 1.0 % uncertainty for f_IS_. This work demonstrates that the measurement uncertainty in f_IS_ is the major driver of the uncertainty in glucose yields from enzymatic hydrolysis and ethanol yield from xylose in fermentation. Clearly there is a need for an improved f_IS_ measurement in terms of both accuracy and precision. We are currently performing experiments to address this issue.

Additionally, we note the difference in the measurement uncertainties between pretreatment relative to enzymatic hydrolysis and fermentation (Figures 3, 4 and 5). These results suggest that uncertainty in pretreatment yield is not overwhelmed by the f_IS_ measurement, but rather is affected by a set of mass flows, composition measurements, and f_IS_ without a single parameter being dominant. This is in part because pretreatment depends on mass flow parameters, as it is operated in a continuous fashion whereas enzymatic hydrolysis and fermentation are operated in batch mode. In addition, the yields from enzymatic hydrolysis and fermentation require two f_IS_ measurements, whereas the pretreatment unit yield only requires a single f_IS_ measurement.

### MESP uncertainty

The Monte Carlo analysis of the TE model provided a distribution of MESP values with a mode of $2.17/gal and a 95 % confidence interval of $2.09-2.39/gal (Figure 6). We interpret this confidence interval as the uncertainty associated with estimation of the MESP due to uncertainty in primary measurements entered in to the process model. These measurements come from experiments designed to determine yields for the three major unit operations: pretreatment, enzymatic hydrolysis, and fermentation. While the utility of a TE model is to compare and contrast different technical and economic scenarios, any results from such studies should be viewed in the context of this work; the minimum significant difference in MESP predictions is half the 95 % confidence interval, or approximately $0.15/gal.

Almost 30 % of the total MESP uncertainty is associated with fermentation, 60 % from enzymatic hydrolysis, and 5 % each for pretreatment and feedstock composition. The large influence of enzymatic hydrolysis yield uncertainty is due to a combination of two factors: the sensitivity of MESP to this yield (Figure 2) and the magnitude of the uncertainty of this measured value (Figure 4). This suggests that future efforts to improve analytical methods should focus on improving yield calculations for enzymatic hydrolysis first, then fermentation, and lastly pretreatment. Since the f_IS_ measurement is the key driver of uncertainty in these yield measurements, it is likely that efforts to reduce the uncertainty of f_IS_ measurement will provide the most benefit.

## Conclusions

We have demonstrated a robust and versatile framework for evaluating the impact of measurement uncertainties on the precision of outputs of a process model for the conversion of lignocellulosic biomass to ethanol. This generalized framework can be readily deployed to other TE models for various biorefinery scenarios, and will aid in the prioritization of research efforts by focusing attention on the most important variables for reduction of measurement uncertainty. Here, we first used a sensitivity analysis to identify the input parameters in the process model that have the greatest impact on the MESP calculated by the overall TE model. These input parameters include feedstock composition and calculated yields for the three unit operations (pretreatment, enzymatic hydrolysis, and fermentation). We identified the experimental measurements that determine the precision of these calculated yields and then performed an uncertainty analysis on these yields. We decomposed the results to highlight the individual contributions of each primary measurement involved in a given yield calculation. We then performed a Monte Carlo analysis of the overall techno-economic (TE) model using normal distributions of the key input parameters that have standard deviations equal to the uncertainties of these parameters. The Monte Carlo analysis allowed us to estimate the uncertainty in MESP due solely to uncertainties in experimental data used to calculate yields in the three major unit operations. We estimate the uncertainty in the MESP to be $0.15/gal of ethanol. This sets a lower bound on the error bars of the TE model; competing model scenarios with MESP estimates that differ less than this value are essentially equivalent.

## Methods

The overall methodology, shown in Figure 1, includes a preliminary sensitivity analysis to determine how the MESP value changes in response to changes in each feedstock composition and process yield value, the derivation of analytical expressions to calculate the uncertainty of those yields based on uncertainty in primary measurement values, and a Monte Carlo analysis of the MESP to provide an estimate of the uncertainty in calculated MESP values.

### Sensitivity analysis of TE model

 A normalized global sensitivity calculation relates the relative change in MESP to the relative change of a given variable. Mathematically, it is defined as:

(2) $$\text{Normalized}\;\text{Sensitivity}=\frac{\left(\Delta MESP/MESP\right)}{\Delta X/X}=\frac{d\ln MESP}{d\ln X}$$

where *X* is the variable subjected to perturbation. In an alternative form, the normalized sensitivity is equal to the slope of the line relating ln(MESP) to the ln(*X*). The results are interpreted as follows: if the resulting sensitivity value is 1.5, it means that a 1 % change in variable *X* results in a 1.5 % change in relative MESP. If the sensitivity is positive, then the relative MESP increases when *X* increases; if sensitivity is negative, the MESP decreases when *X* increases. The range of perturbation is set to be 15 % above and below the base case value of *X*. The “base case” refers to the set of variable values that most accurately describes the state of the process at the time of analysis. For this work, the base case values were taken from the NREL Design Report default cases [39]. In some cases, multiple reactions use the same starting material, so the perturbation range must be modified to maintain a physically-relevant result (i.e. to maintain mass closure). For example, in the fermentation unit, there is a group of reactions that describe the conversion of glucose to ethanol. The total yield of these reactions is 99.7 %, so certain reaction yields cannot be increased to 15 % above their base case values without producing an unphysical result. In this case, the range of perturbation is −15 % to 0 % of the base case value.

### Uncertainty analysis of yield expressions

The uncertainty analysis presented here is based on the approach recommended by the International Standards Organization (ISO) [41]. We estimate the uncertainty of a calculated output variable by identifying all sources of uncertainty in the measured input variables, specifying their relationship to the output variable, estimating their uncertainty, and then calculating the uncertainty of the output variable using the following expression:

(3) $$U_{e}^{2}={\sum_{i=1}^{n}\left(\frac{\partial e}{\partial k_{i}}\right)}^{2}U_{k_{i}}^{2}$$

where *e* is the calculated output variable, *k* is a measured “input” variable, and *i* is an index for the input variables. Therefore, *k*_*i*_ is the *i*^*th*^ measured input variable used to calculate the output variable *e*, and *U*_*ki*_ is the uncertainty in this measurement. *U*_*e*_ is the uncertainty of *e*. In general, the expression for yield uncertainty is derived from the yield expression itself. The yield expression will contain primary measurements, derived quantities, and constants. A derived quantity is a term that is not measured explicitly; it is calculated from other primary measurements. The uncertainty of a derived quantity is also calculated according to Equation 3. This uncertainty is then used in the yield uncertainty calculation. A sample calculation is provided in the Supplementary Material (Additional File 5).

### Monte Carlo analysis of MESP from TE model

The uncertainties in the calculated yield values and the feedstock composition were used in a Monte Carlo analysis of the economic model to produce an uncertainty for the MESP estimate. A total of 5,000 combinations of input parameters have been tested using the baseline values from the design report and normal distributions of the important variables with standard deviations set to the uncertainties calculated in this work. The distributions have been confined to +/− two standard deviations. The sampling distributions have been constrained such that the distributions of yield values have been limited to values less than 100 %. The details of these distributions are shown in Table 1.

**Table 1:** Input parameters to the Monte Carlo analysis of minimum ethanol selling price (MESP) uncertainty

**Parameter**	**Base value (%)**	**Maximum value (%)**	**Uncertainty**	**Source**
**Feedstock composition**				
Glucan	35.1	--	0.53	[34] Table 1
Xylan	19.5	--	0.34	
Lignin	15.8	--	0.17	
**Pretreatment yield**				
Xylan to Xylose	90.0	95.0	2.7	this work
Arabinan to Arabinose	90.0	95.0	7.6	
**Enzymatic Hydrolysis yield**				
Cellulose to Glucose	90.0	95.0	5.2	this work
**Fermentation yield**				
Glucose to Ethanol	95.0	100.0	3.00	this work
Xylose to Ethanol	85.0	95.0	3.00	

The base values for all parameters were taken from the 2011 Design Report [39]. Uncertainties for feedstock composition were taken from [34]. Uncertainties in calculated yield values are from this work. Using normal distributions of these feedstock composition and calculated yield values, we estimate a mode of $2.17/gal for the MESP with an uncertainty of $0.15/gal.

## Abbreviations

MESP: Minimum Ethanol Selling Price; TE: Techno-economic; f_IS_: Fraction of insoluble solids.

## Competing interests

The authors declare no financial or non-financial competing interests.

## Authors’ contributions

EJW, CJS, and GTB conceived of and participated in the design of the study. SST, TS, and KKJ performed sensitivity, uncertainty, and Monte Carlo analyses, and wrote an internal report that served as the basis of this manuscript as part of the MIT Practice School. KJV and GTB provided oversight during the Practice School, and KJV developed the first draft of the manuscript. DH, CJS, and GTB provided technical support during the Practice School. DH performed sensitivity and Monte Carlo analyses for the final version of the manuscript. EJW, GTB, and KJV edited several versions of the manuscript. All authors read and approved the final manuscript.

## Supplementary Material

Additional file 1 **Information about Uncertainty Input Data.** This spreadsheet documents all of the measured uncertainties used as inputs for this uncertainty analysis and their experimental sources. Click here for file

Additional file 2 **Pretreatment Uncertainty Calculations.** This spreadsheet documents the calculation of uncertainty values in the pretreatment operation of the process. It highlights the input uncertainties and relevant parameters, as well as the resulting uncertainties presented in several formats. Click here for file

Additional file 3 **Enzymatic Hydrolysis Uncertainty Calculations.** This spreadsheet documents the calculation of uncertainty values in the enzymatic hydrolysis operation of the process. It highlights the input uncertainties and relevant parameters, as well as the resulting uncertainties presented in several formats. Click here for file

Additional file 4 **Fermentation Uncertainty Calculations.** This spreadsheet documents the calculation of uncertainty values in the fermentation operation of the process. It highlights the input uncertainties and relevant parameters, as well as the resulting uncertainties presented in several formats. Click here for file

Additional file 5 **Example of Uncertainty Expression Derivation.** This document explains in detail the derivation of an analytical expression for yield uncertainty, starting with the yield expression itself. The example discussed is the yield of glucose from cellulose. This reaction takes place in the enzymatic hydrolysis unit. The yield itself is calculated from two measured values and one derived quantity. The uncertainties of the measured values are measured experimentally. The uncertainty of the derived quantity is calculated from the uncertainties in the primary measurements that are used to calculate the value of the derived quantity. The final analytical expressions are presented, as well as intermediate steps. Click here for file
