# Supplementary material for: Uncertainty in techno-economic estimates of cellulosic ethanol production due to experimental measurement uncertainty
Source: Biotechnol Biofuels. 2012 Apr 17;5:23. doi: 10.1186/1754-6834-5-23 (PMC3503575; doi:10.1186/1754-6834-5-23)
Supplement: Additional file 5 — Example of Uncertainty Expression Derivation. This document explains in detail the derivation of an analytical expression for yield uncertainty, starting with the yield expression itself. The example discussed is the yield of glucose from cellulose. This reaction takes place in the enzymatic hydrolysis unit. The yield itself is calculated from two measured values and one derived quantity. The uncertainties of the measured values are measured experimentally. The uncertainty of the derived quantity is calculated from the uncertainties in the primary measurements that are used to calculate the value of the derived quantity. The final analytical expressions are presented, as well as intermediate steps. [file 1754-6834-5-23-S5.pdf]

Examples of Uncertainty Expression Derivation:

Uncertainty of yield of glucose from cellulose in the enzymatic hydrolysis unit

The percent yield of glucose from cellulose is calculated from the following expression:

$$Y_{C\_G} = \frac{w_G}{f_{IS} \times 1000 \times f_{PG} \times \gamma} \times 100\% \qquad (1)$$

The terms in the yield expression are defined in the table below.

Term	Description (Units)
Y_{C_G}	Yield of glucose from cellulose (percent yield)
w_G	Weight fraction of glucose in final solution (g glucose per kg of total solution)
f_{IS}	Fraction insoluble solids (mass fraction)
f_{PG}	Fraction of glucan in pretreated liquor (mass fraction)
γ	A (constant) conversion factor that corrects for the change in mass due to the addition of water to the glycosidic bonds during hydrolysis of glucan (dimensionless)

The factor of 1000 converts grams to kilograms. The term "f_{PG}" is the mass fraction of feedstock that is cellulose. It is assumed that all of this cellulose is insoluble, so the f_{IS} term is required to convert from between the cellulose fraction and the mass fraction of (soluble) glucose.

The mass fraction of glucose is a "derived" quantity, meaning that it is a term calculated from other measurements. This calculation is shown below.

$$w_X = \frac{(1 - f_{IS})(c_{Gf} - c_{Gi})}{\rho_L} \qquad (2)$$

Term	Description (Units)
w_G	Weight fraction of glucose in final solution (g glucose per kg of total solution)
f_{IS}	Fraction insoluble solids (mass fraction) in the slurry after enzymatic hydrolysis
c_{Gf}	"Final" concentration of glucose in slurry after enzymatic hydrolysis (g/L)
c_{Gi}	"Initial" concentration of glucose in slurry, before hydrolysis (g/L)
ρ_L	Density of pretreated liquor (g/mL)

Recall that the uncertainty in any term is the sum of the uncertainties of its components multiplied by the relevant partial derivative.

$$U_{Y_{C\_G}}^2 = \sum_{i=1}^{n} \left(\frac{\partial Y_{C\_G}}{\partial k_i} \right)^2 U_{k_i}^2 \tag{3}$$

Specifically, the uncertainty in the yield of glucose from cellulose is calculated from the expression below.

$$\begin{aligned} U_{Y_{C\_G}}^2 = & \left(\frac{100}{1000 f_{IS} f_{PG} \gamma} \right)^2 U_{w_G}^2 + \left(\frac{100 w_G}{1000 f_{IS} f_{PG} \gamma^2} \right)^2 U_{\gamma}^2 \\ & + \left(\frac{100 w_G}{1000 f_{IS}^2 f_{PG} \gamma} \right)^2 U_{f_{IS}}^2 + \left(\frac{100 w_G}{1000 f_{PG}^2 f_{IS} \gamma} \right)^2 U_{f_{PG}}^2 \end{aligned} \tag{4}$$

Since the mass fraction of glucose in the solution (w_G) is a derive quantity, its uncertainty is not measured. Rather its uncertainty is calculated from an analogous sum of terms, shown below.

$$U_{w_G}^2 = \left(\frac{(1 - f_{IS})}{\rho_L} \right)^2 U_{c_{Gf}}^2 + \left(-\frac{(1 - f_{IS})}{\rho_L} \right)^2 U_{c_{Gi}}^2 + \left(\frac{-(c_{Gf} - c_{Gi})}{\rho_L} \right)^2 U_{f_{IS}}^2 + \left(-\frac{(1 - f_{IS})(c_{Gf} - c_{Gi})}{\rho_L^2} \right)^2 U_{\rho_L}^2 \tag{5}$$

Plugging Equation 5 in to the mass fraction term in Equation 4 results in an expression of the yield uncertainty that is a function of measurement uncertainties and the “base” values of certain variables, as well as any relevant proportionality constants.
